# Autistic traits, resting-state connectivity, and absolute pitch in professional musicians: shared and distinct neural features

**DOI:** 10.1186/s13229-019-0272-6

**Published:** 2019-05-02

**Authors:** T. Wenhart, R. A. I. Bethlehem, S. Baron-Cohen, E. Altenmüller

**Affiliations:** 1 Institute of Music Physiology and Musicians’ Medicine, University for Music, Drama and Media, Hannover, Germany; 2 Center for Systems Neuroscience, Hannover, Germany; 3 Autism Research Center, Department of Psychiatry, University of Cambridge, Cambridge, UK

**Keywords:** Absolute pitch, Autistic traits, Brain networks, Graph theory, Musicians, Electroencephalography

## Abstract

**Background:**

Recent studies indicate increased autistic traits in musicians with absolute pitch and a higher proportion of absolute pitch in people with autism. Theoretical accounts connect both of these with shared neural principles of local hyper- and global hypoconnectivity, enhanced perceptual functioning, and a detail-focused cognitive style. This is the first study to investigate absolute pitch proficiency, autistic traits, and brain correlates in the same study.

**Sample and methods:**

Graph theoretical analysis was conducted on resting-state (eyes closed and eyes open) EEG connectivity (wPLI, weighted phase lag index) matrices obtained from 31 absolute pitch (AP) and 33 relative pitch (RP) professional musicians. Small-worldness, global clustering coefficient, and average path length were related to autistic traits, passive (tone identification) and active (pitch adjustment) absolute pitch proficiency, and onset of musical training using Welch two-sample tests, correlations, and general linear models.

**Results:**

Analyses revealed increased path length (delta 2–4 Hz), reduced clustering (beta 13–18 Hz), reduced small-worldness (gamma 30–60 Hz), and increased autistic traits for AP compared to RP. Only clustering values (beta 13–18 Hz) were predicted by both AP proficiency and autistic traits. Post hoc single connection permutation tests among raw wPLI matrices in the beta band (13–18 Hz) revealed widely reduced interhemispheric connectivity between bilateral auditory-related electrode positions along with higher connectivity between F7–F8 and F8–P9 for AP. Pitch-naming ability and pitch adjustment ability were predicted by path length, clustering, autistic traits, and onset of musical training (for pitch adjustment) explaining 44% and 38% of variance, respectively.

**Conclusions:**

Results show both shared and distinct neural features between AP and autistic traits. Differences in the beta range were associated with higher autistic traits in the same population. In general, AP musicians exhibit a widely underconnected brain with reduced functional integration and reduced small-world property during resting state. This might be partly related to autism-specific brain connectivity, while differences in path length and small-worldness reflect other ability-specific influences. This is further evidenced for different pathways in the acquisition and development of absolute pitch, likely influenced by both genetic and environmental factors and their interaction.

## Background

Autism spectrum disorders or conditions (henceforth “autism”) are more common in people with mathematical [1], visuospatial [2], musical [3], or “savant” abilities [4], e.g., rapid mental mathematical calculation [5, 6], calendar calculation [7], or extreme memory [8, 9]. Autism, a set of neurodevelopmental condition, is characterized by social and communication difficulties, alongside unusually repetitive behaviors and unusually narrow interests [10], sensory hypersensitivity, and difficulties in adjusting to unexpected change (DSM-5, APA 2013).

Absolute pitch (AP), the ability to name or produce a musical tone without the use of a reference tone [11], is a common special ability in professional musicians with a proportion of up to 7–25% [12–14] but less than 1% [15] in the general population. AP is an excellent model for the investigation of a joint influence of genetic and environmental factors on the brain and on human cognitive abilities [16]. Even if the ability is relatively rare, various studies suggest that the ability might be distributed more gradually than expected [17–19]. Partial AP ability seems to be common in professional musicians, who might in conjunction with good relative pitch strategies (interval judgements) yield moderate to good results in absolute pitch tests [18]. An influence of age of onset of musical training [20–22], ethnicity [12, 14, 22], and type of musical education (label to fixed pitch vs. label to interval, unfixed to pitch) techniques [12]) suggest environmental aspects in the acquisition of AP. In contrast, AP often clusters in families, genetically overlaps with other familial aggregated abilities (e.g., synesthesia [23]), and has a higher proportion in autistic people [3, 7, 24–29] and in Williams syndrome [30, 31], both strongly genetic conditions [32–39]. Remarkably, some studies on musically untrained children with and without ASC could even show increased long-term memory for pitch [3, 27, 28] in ASC children. Finally, a sensitive or critical period before the age of seven is considered due to the importance of the early onset of musical training [14, 16, 20, 40–43]. Relative pitch abilities, i.e., the ability to perceive equal intervals between musical tones as similar and to be able to judge the pitch height of tones relative to each other, is very common in the general population. RP abilities also show high variability in absolute pitch possessors [18, 44–46]. Musically trained people, however, often exhibit more explicitly developed RP abilities (e.g., verbal labeling of musical intervals to a similar proficiency as tone labeling of absolute pitch possessors) than less musically trained or musically untrained people [18].

Recently, two studies have given evidence for heightened autistic traits in musicians with AP [47, 48]. Both AP and autism are associated with similarly altered brain connectivity in terms of the relation between hyper- and hypoconnectivity [41, 49–58]. The theory of veridical mapping [7] tries to explain absolute pitch, synesthesia, and other abilities like hyperlexia, frequently seen in autistic people or in savant syndrome, with the neurocognitive mechanism of associating homolog patterns of two perceptual or cognitive structures (veridical mapping). According to this framework, an enhanced low-level perception [59, 60] and an increased ability to detect patterns (“systemizing” [61]) are associated with regional hyper- as well as global hypoconnectivity in absolute pitch [49, 51, 62–67] and autism [50, 52, 54, 68]. It is also noteworthy that autism and abilities like absolute pitch share excellent attention to detail [40, 69] and a shift in the direction of higher segregation with reduced integration in the brain [69]. Investigating disconnection syndromes or integration deficit disorders, as well as phenomena with similar brain network characteristics, may therefore provide insights into the variability of brain network structure and function and its relation to perception, cognition, and behavior.

The present study tests if and to what extent AP and autistic traits share the same neurophysiological network connectivity. To our knowledge, this study is the first to investigate (1) the relation of pitch adjustment ability (active absolute pitch; in contrast to (passive) pitch identification) and brain as well as behavioral correlates; (2) the relation of AP ability, autistic traits, and functional brain connectivity within one study; and (3) graph theoretical network parameters in AP during resting-state electroencephalography. We used graph theoretical analysis [70, 71] of resting-state EEG data to estimate differences in global network structure of the brain. We analyzed three graph theoretical network parameters reflecting segregation (average clustering coefficient) and integration (average shortest path length) and the so called small-worldness (a combination of clustering and path length) [70, 71]. To our knowledge, this is also the first study investigating the global average connectivity parameters over the whole brain between AP and RP (relative pitch) musicians, while prior studies [49, 51] have focused on parameters for single regions (e.g., degree, single node clustering, and single node characteristic path length). We expected higher autistic traits, higher path length (reduced integration), and lower clustering (underconnectivity) for AP and an interrelation among those variables. Further, we expected these differences to specifically occur in low- (delta, theta) vs. high-frequency (beta) ranges for integration vs. segregation, respectively.

## Methods

### Participants

Thirty-one AP musicians (16 female) and 33 RP musicians (15 female) participated in the study. One male RP participant had to be excluded from the EEG analysis because of missing EEG data. Participants were recruited via an online survey using UNIPARK software (https://www.unipark.com/) and primarily were students or professional musicians at the University for Music, Drama and Media, Hanover. Four APs and two RPs were amateur musicians. An online pitch identification screening (PIS) consisting of 36 categorical, equal-tempered sine waves in the range of three octaves between C4 (261.63 Hz) and B6 (1975.5 Hz) was used to allocate the participants to the groups (AP > 12/36 tones named correctly, else RP). Some of our subjects reported to have absolute pitch (from our experience, professional musicians usually know whether they themselves have absolute pitch or not) and performed like absolute pitch possessors in the absolute pitch adjustment test in the lab (see the “Pitch adjustment test”section), despite their comparable weak performance in the pitch identification test online. Their weak performance in the online test might have resulted from technical problems of personal subjects’ devices to present the tones (reported during personal communication). The online test was also performed under uncontrolled experimental condition. Additionally, 2 participants had 13 and 21 correct answers, respectively, in the online pitch-naming test but performed weakly in the pitch adjustment test (and reporting not to have absolute pitch) and were re-assigned to the RP group. Musicians without “real” absolute pitch occasionally yield above chance results in pitch-naming tests, which might be caused by the use of experience-based strategies in the test (e.g., having pitch of empty strings in string players or starting tones of famous melodies as comparison in mind) [18].

There is currently no consensus about a cutoff in terms of percentage of tones named correctly to be defined as absolute pitch possessor. To verify the decision for the cutoff, we compared performances in pitch naming and adjustment by inspecting scatter plots. For the clear difference between AP and RP in the pitch adjustment test, where RP strategies do not seem to help (personal reports after test), we decided to take a cutoff of 12/36 tones named correctly and not a higher, e.g., 50% or 80% cutoff. However, the cutoff for the pitch-naming test may not reflect a suitable cutoff for other samples. Four AP were non-native German speakers and had the choice between a German and an English version of the experiments. One AP reported taking mirtazapine. None of the participants reported any history of severe psychiatric or neurological conditions. The AP group consisted of 15 pianists, 9 string players, 3 woodwind instrument players, 2 singers, and 2 brass players; the RP group consisted of 13 pianists, 4 string players, 6 woodwind instrument players, 3 bassists/guitarists/accordionists, 3 singers, 1 drummer, and 3 brass players. Handedness was assessed by Edinburgh Handedness Inventory [72]; one AP was left handed, all other APs were consistently right handed, three RPs were left handed, and two RPs were ambidextrous. This study was approved by the local Ethics Committee at the Medical University Hannover. All participants gave written consent.

### Setting

The study was divided into three parts: the online survey and two appointments in the lab at the Institute for Music Physiology and Musicians’ Medicine of the University for Music, Drama and Media, Hannover. While the online survey was used for the pitch identification screening and diagnostic as well as demographic questionnaires (see below), general intelligence, musical ability, pitch adjustment ability, and resting-state EEG were assessed in the lab (see Table 1). Four further experiments were conducted within the same two sessions at the lab and are reported elsewhere [73, 74]. Raven’s Standard Progressive Matrices [75] and “Zahlenverbindungstest” (ZVT, [76]) were used to assess general non-verbal intelligence and information processing speed, respectively. Musical ability and musical experience were controlled for with the use of AMMA (Advanced Measures of Music Audiation [77]), Musical Sophistication Index (GOLD-MSI, [78]), and estimated total hours of musical training within life span (house intern online questionnaire).

**Table 1:** Participants’ characteristics

	AP (*n* = 31)			RP (*n* = 33)			*t* test
	Mean	SD	Range	Mean	SD	Range	
Age (years)	25.13	9.2	17–58	24.0	7.02	17–57	*t*(56.1) = − 0.549; *p* = 0.585
SPM (IQ)	110.4	16.4	73–132.25	114.41	13.14	86.5–134.5	*t*(57.5) = 1.073; *p* = 0.288
ZVT (IQ)	120.76	13.14	101.5–145	120.61	13.69	97–143.5	*t*(61.9) = − 0.045; *p* = 0.964
Hours main instrument (h)	11,961.4	9212	1642.5–39,785	13,735.61	17,125.89	1606–77,617.25	*t*(49.7) = 0.520; *p* = 0.605
AMMA total	64.74	6.26	53–78	63.244	7.03	46–76	*t*(61.8) = − 0.90; *p* = 0.370
AMMA rhythmic	32.81	2.82	28–39	31.97	3.22	23–37	t(61.7) = 0.272; *p* = 0.2721
AMMA tonal	31.9	3.74	25–39	30.27	3.8	22–37	t(61.9) = −1.728; *p* = 0.089
MSI	208.65	17.59	161–234	210.79	15.12	185–246	t(59.3) = 0.521; *p* = 0.604
PIS	28.5	6.03	15–36	5.30	4.33	0–21*	*t(52.2) = − 17.37; p < 2.2e−16*
Starting age (years)	5.97	2.97	2–17	7.12	2.22	3–12	*t*(55.4) = 1.751; *p* = 0.086

Age, non-verbal IQ (SPM, IQ values), information processing capacity (ZVT, IQ values), musical training (total hours during life span on main instrument in hours), musicality (AMMA total, raw score on test; AMMA tonal, tonal raw score; AMMA rhythmic, rhythmic raw score; MSI, questionnaire, sum score; higher values indicate higher musicality), and online pitch identification screening (PIS, sum of correctly named tones) for each group. No group differences apart from performance on pitch-naming test (PIS) were found

*Two RPs reported not having absolute pitch but reached a screening score of 13 and 21, respectively. Because of this and their weak performance in the pitch adjustment test, the subjects were assigned to the RP group. Significant group differences are indicated in italics

### Experiments and material

#### Pitch adjustment test

Absolute pitch ability was measured by using two different absolute pitch tests: the pitch identification screening (PIS) during the online survey mentioned above and a pitch adjustment test (PAT) based on Dohn et al. [79]. Participants were given a maximum of 15 s to adjust the frequency of a sine wave with random start frequency (220–880 Hz, 1 Hz steps) and told to try to hit the target note (letter presented central on PC screen, e.g., “F#/Gb”) as precisely as possible without the use of any kind of reference. Online pitch modulation was programmed according to Dohn et al. [79] and provided by turning a USB controller (Griffin PowerMate NA16029, Griffin Technology, 6001 Oak Canyon, Irvine, CA, USA). Resolution of the PowerMate was set to 10 cents vs. 1 cent (if pressed during turn of the wheel) for individual choice between rough and fine tuning. To confirm their answer, participants were instructed to press a button on a Cedrus Response Pad (Response Pad RB-844, Cedrus Corporation, San Pedro, CA 90734, USA) to automatically proceed with the next trial. If no button was hit, the final frequency after 15 s was taken. In both cases, the intertrial interval (ITI) was set to 3000 ms. The total test consisted of 108 target notes, presented in a semi-random order in 3 blocks of 36 notes each (3 × 12 different notes per block) with individual breaks between the blocks. The final or chosen frequencies of each participant were compared to the nearest target tone (< 6 semitones/600 cent), as participants were allowed to choose their octave of preference. EEG was measured during the PAT but will be reported elsewhere. For each participant, the mean absolute derivation (MAD (1), [79]) from target tone:

1 $$ \mathrm{MAD}=\frac{\sum \limits_{i=1}^{N_{\mathrm{adjustment}}}\mid {C}_i\mid }{N_{\mathrm{adjustment}}} $$

is calculated as the mean of the average absolute deviations *c*_*i*_ (2) of the final frequencies to the target tone (referenced to a 440 Hz equal-tempered tuning).

MAD reflects the pitch adjustment accuracy of the participants. The consistency of the pitch adjustments, possibly reflecting the tuning of the pitch template [79], is then estimated by taking the standard deviation of the absolute deviations (2).

2 $$ \mathrm{SDfoM}=\sqrt{\frac{\sum \limits_{i=1}^{N_{\mathrm{adjustment}}}{\left({C}_i-\mathrm{MAD}\right)}^{{}^2}}{N_{\mathrm{adjustment}}-1}} $$

For regression analyses (see below), we performed a z-standardization of the MAD (Z_MAD, (3)) and SDfoM (Z_SDfoM, (4)) values relative to the mean and SD of the non-AP group, as originally proposed by Dohn et al. [79].

3 $$ \mathrm{Z}\_{\mathrm{MAD}}_i=\frac{{\mathrm{MAD}}_i-\mu {\left(\mathrm{MAD}\right)}_{\mathrm{Non}-\mathrm{AP}}}{\sigma {(MAD)}_{\mathrm{Non}-\mathrm{AP}}} $$

4 $$ \mathrm{Z}\_{\mathrm{SDfoM}}_i=\frac{{\mathrm{SDfoM}}_i-\mu {\left(\mathrm{SDfoM}\right)}_{\mathrm{Non}-\mathrm{AP}}}{\sigma {\left(\mathrm{SDfoM}\right)}_{\mathrm{Non}-\mathrm{AP}}} $$

#### Autistic traits

Autism traits were assessed during the online survey using a standardized Adult Autism Spectrum Quotient (AQ, [80]; German version by C.M. Freiburg, available online: https://www.autismresearchcentre.com/arc_tests). It consists of 50 items within 5 subscales (attention to detail, attention switching, imagination, social skills, and communication). One point is given for each item with a mildly or strongly agreement with the autistic-like symptoms (half of the items were negatively poled; the maximum AQ score therefore is 50).

#### EEG resting state

EEG resting-state data was acquired immediately before the PAT at the beginning of the experimental session using 28 scalp electrodes (sintered silver/silver chloride; Fp1, Fp2, F3, F4, FC3, FC4, C3, C4, CP3, CP4, P3, P4, F7, F8, FT7, FT8, T7, T8, TP7, TP8, P7, P8, O1, O2, Oz, Fz, Cz, Pz) placed according to the international extended 10–20 System with an electrode cap by EASYCAP (EASYCAP GmbH, Herrsching, Germany; http://www.easycap.de). A 32-channel SynAmps amplifier (Compumedics Neuroscan, Inc., Charlotte, NC, USA) and the software Scan 4.3 (Compumedics Neuroscan) were used to record the data. The remaining two bipolar channels were used for the vertical and horizontal electrooculogram with electrodes placed above and below the right eye and approximately 1 cm outside of the outer canthus of each eye, respectively. Two further electrodes were placed on the left and right mastoids as a linked reference. The ground electrode was placed between the eyebrows directly above the nasion on the forehead. Abralyth 2000 abrasive chloride-free electrolyte gel (EASYCAP GmbH, Herrsching, Germany; http://www.easycap.de) was used to keep impedances below 5k Ω. Participants were seated in a comfortable chair in front of a PC screen and were instructed to let their mind wander around while looking at a fixation cross (eyes open resting state, EO) or keeping their eyes closed (eyes closed resting state, EC) for 5 min each. All participants underwent both resting-state conditions and started with the 5-min eyes open condition followed by the 5-min eyes closed. Start (button press) and end of the resting-state period were programmed within PsychoPy [81] by sending triggers via a parallel port to the EEG system. A sampling rate of 1000 Hz was used combined with an online bandpass filter between 0.5–100 Hz and a Notch filter at 50 Hz. EEG was recorded in AC (alternating current) mode and with a gain of 1000.

### EEG preprocessing and analysis

#### Preprocessing

All preprocessing steps were conducted using MATLAB (MATLAB Release 2014a, MathWorks, Inc., Natick, MA, USA) and the toolboxes EEGLAB [82] and FieldTrip [83]. EEG raw data was first re-sampled to 512 Hz sampling rate and bandpass filtered to 1–100 Hz. Artifact removal was administered using both raw data inspection of continuous data and independent component analysis (ICA, algorithm: binica) within EEGLAB for each participant’s data individually. ICA components containing vertical or horizontal eye movements, blinking, heartbeat, muscular activity, or other artifacts were removed from the data by inverse ICA. After that, segments still containing the abovementioned artifacts were removed manually. Defective or highly noisy electrodes were interpolated using spherical interpolation [84] implemented in EEGLAB (5 participants, 1–2 electrodes each). All statistical analyses were repeated under the exclusion of participants with interpolated electrodes as well as non-native German speakers and the participant which reported to take mirtazapine. Direction and significance of effects were not affected by the exclusions; therefore, all participants were included into the final analyses. Afterwards, the artifact-clean data was exported to FieldTrip for the connectivity and network analysis (next steps).

#### Connectivity—weighted phase lag index

The calculation of functional connectivity was done using MATLAB scripts (see: https://github.com/rb643/fieldtrip_restingState/blob/master/rb_EEG_Conn.m). First, 4-s epochs (non-overlapping) were extracted from the artifact-clean data. Epochs that still contained artifacts were removed for each subject (0–5 epochs of 75 epochs per subject and resting-state condition). Second, multi-taper Morlet fast Fourier transformation was used to extract frequency bands (delta 2–4 Hz, theta 4–7 Hz, alpha 7–13 Hz, beta 13–30 Hz, gamma 30–60 Hz). For delta and theta, a single taper (Hanning window) was used. On the contrary, for alpha, beta, and gamma, multiple tapers (discrete prolate spheroidal sequences, DPSS) were taken. During the multi-tapering of alpha, beta, and gamma, spectral smoothing was applied (+ − 1, 2, 4 Hz, respectively). Finally, pairwise connectivity values for each electrode site were calculated per participant and stored in a connectivity matrix for each frequency band separately. Weighted phase lag index [85] was chosen as the connectivity measure, as phase-based connectivity measures compared to coherence and phase synchronization measures are less sensitive to volume conduction in the brain [86, 87] (cited by [88]), i.e., spurious connectivity between the two regions of interest caused by a common source of activity or a common reference [89] and usually leads to connectivity values with phase lags of zero or pi (if the two sites are on opposite sides of the dipole) [90]. PLI (5) is an index that quantifies the asymmetry of the distribution of instantaneous phase-differences ∆Φ between the signals *x* and *y*, by averaging the sign (sgn) of the imaginary components (imag) of the cross-spectrum (*S*_xyt_) at time point *t* [90].

5 $$ {\mathrm{PLI}}_{xy}=\mid {n}^{-1}\sum \limits_{t=1}^n\operatorname{sgn}\left(\mathrm{imag}\left({S}_{xy t}\right)\right)\mid $$

The distribution is centered around 0 mod pi; therefore, an asymmetric distribution shows a non-zero phase lag. Stam et al. [89] argue that a non-zero phase lag cannot be caused by a volume conduction or a common reference, as the latter works instantaneously. PLI takes values between 0 and 1, where 0 indicates no phase coupling (or a coupling with a 0 mod pi phase difference) and 1 indicates a perfect coupling at the phase lag of ∆Φ. Because of the absolute values taken in Eq. (1), PLI does not give information about which signal is leading [89]. PLI has been shown to be superior in detecting true synchronization and in being less influenced by common source activity and electrode montage systems than phase coherence (PC, [91]), both in computer simulations and on real EEG and MEG data [89]. Furthermore, PLI exhibits a similar amount of long- to short-distance connections in an investigation of beta band coupling in the Alzheimer data [89, 92] which was shifted towards short-range connections implying volume conduction when using PC [89]. As the aim of this paper is to compare graph theory-based network measures that especially quantify segregation versus integration in the brain (see the “Network analysis—graph theory” section), the use of PLI is to be preferred to prevent the distortion of the network parameters by volume conduction [93, 94]. The extension of PLI, weighted PLI (wPLI, (6) [85]),

6 $$ {\mathrm{wPLI}}_{xy}=\frac{n^{-1}\sum \limits_{t=1}^n\mid \mathrm{imag}\left({S}_{\mathrm{xyt}}\right)\mid \operatorname{sgn}\left(\mathrm{imag}\left({S}_{\mathrm{xyt}}\right)\right)}{n^{-1}\sum \limits_{t=1}^n\mid \mathrm{imag}\left({S}_{\mathrm{xyt}}\right)\mid } $$

weights the obtained phase leads or lags by the magnitude of the imaginary component (imag) of the cross-spectrum (*S*_xyt_). This reduces the influence of additional noise sources [85, 90]. Weighted phase lag index [85] therefore is an advancement of phase lag index (PLI, [89]) and a suitable measure to detect the true connectivity between the regions of interest [89], as it ignores zero- and pi-phase lag.

#### Network analysis—graph theory

Graph network analyses were conducted using Brain Connectivity Toolbox (BCT, [95]) in MATLAB. Graph theory is a branch of mathematics that deals with the abstract representation of networks as graphs, i.e., a system of *n* nodes and *k* edges (connections) between the nodes. Increasingly, network science is being applied to a range of neuroanatomical and physiological data (e.g., [49, 51, 96–101]) and at different scales of interest (e.g., neurons/populations of neurons, cortical areas, electrode sites; see [70, 95, 102–104] for an overview). In the present study, the pairwise connectivity measures for each frequency band and participant were stored in a 28 × 28 (channel by channel) matrix. Therefore, electrode sites are defined as the nodes and wPLI indexes of the electrode pairs within the matrix as edges. This was done in two steps: First, to construct adjacency matrices for graph analyses, minimal spanning tree (MST: [101]) was used as the threshold starting point for building binary networks at various densities. The density of a network relates to the fraction of edges present in the network compared to the maximum possible number of edges. MST was chosen to ensure that across participants, we were comparing network with similar numbers of nodes (e.g., differential thresholding without MST can lead to unconnected nodes and result to networks of different sizes). Afterwards, we investigated the network properties over a range of densities (0.036, 0.079, 0.106, 0.132, 0.159, 0.212, 0.238, 0.265, 0.291; percent of all possible connections, i.e., ten thresholding levels) by stepwise adding the highest remaining edges. Instead of picking one subjective network threshold, we decided to use equally spaced thresholds within a biologically plausible range to ensure that any findings were consistent across thresholds (see [101]). The process of thresholding itself, however, is still a highly debated issue without consensus. Finally, thresholding leads to ten adjacency matrices, for each frequency band and participant.

To estimate the differences in global network structure of the brain, we analyzed two graph theoretical network parameters reflecting segregation (average clustering coefficient) and integration (average shortest path length) of the brain [70, 71, 105, 106]. It has been shown in a variety of simulations and network analyses of imaging data that the human brain, among other biological systems and animal brains [103, 107], exhibits a small-world architecture [103], which leads to an advantage of efficient information transfer while keeping the anatomical costs low [108, 109]. Compared to the two studies by Jäncke et al. [49] and Loui et al. [51], the present investigation did use network measures averaged over the whole brain and compared to those of a random network, instead of individual values per region. This is advantageous, as the vast variability of individual coherence within a network is reduced to one value per parameter and participant that reflects the small-worldness or efficiency of a brain network relative to a random or chaotic network [70, 92, 95, 102–104]. By definition [70, 107, 108], small-worldness σ (7) is characterized by a *C*, which is much higher than that of a random network (*γ* = *C* real/*C* random > > 1), but has a comparable short path length (*λ* = *L* real/*L* random ≈ 1).

7 $$ \upsigma =\frac{\upgamma}{\uplambda}=\frac{C_{\mathrm{real}}^{\mathrm{w}}/{C}_{\mathrm{random}}^{\mathrm{w}}}{L_{\mathrm{real}}^{\mathrm{w}}/{L}_{\mathrm{random}}^{\mathrm{w}}} $$

Here, the clustering coefficient *C*_*i*_ (8) of a node *i* is defined as the weighted average amount of (9) triangles ${t}_i^w$ around it, i.e., the sum of connections between the neighbors of a node *i* divided by the total amount of possible connections among its neighbors:

8 $$ {C}_i=\frac{1}{n}{\sum}_{i\in N}\frac{2{t}_i^w}{k_i\left({k}_i-1\right)} $$

9 $$ {t}_i^w=\frac{1}{2}{\sum}_{j,h\in N}{\left({w}_{ij}{w}_{ih}{w}_{jh}\right)}^{\frac{1}{3}} $$

The global clustering coefficient (10) of a weighted association matrix *C*^w^ denotes the average clustering coefficient summed over all nodes *i* ∈ *N* in a network and is interpreted as a measure of segregation of the network.

10 $$ {C}^{\mathrm{w}}=\frac{1}{n}{\sum}_{i\in N}{C}_i $$

On the other hand the characteristic path length *L*_*i*_ (11) of a node *i* is defined as the average pairwise distance ${d}_{ij}^w$ (12) between the node *i* and any other node *j* in the weighted (w) network:

11 $$ {L}_i=\frac{1}{n}{\sum}_{i\in N}\frac{\sum_{j\in N,j\ne i}{d}_{ij}^w}{n-1} $$

12 $$ {d}_{ij}^w={\sum}_{a_{uv}\in {g}_{i\leftrightarrow j}^w}f\left({w}_{uv}\right) $$

The global average path length (13) is then calculated by taking the average of the characteristic path length of all nodes *i* ∈ *N* in the network and is interpreted as a measure of integration of the network.

13 $$ {L}^w=\frac{1}{n}{\sum}_{i\in N}{L}_i $$

As both *γ* and *λ* reflect the underlying brain network structure relative to a random network of the same density (and degree distribution) and influence the calculation of small-worldness, we chose to look at these parameters separately, that is, because we were specifically interested in the potentially differential relation of segregation and integration in the brain. Various authors have shown that long-range connections (integration) are more associated with synchronization in low-frequency bands, whereas short-range connectivity is mainly processed within the beta band (e.g., [110]).

### Statistical analysis

All statistical analyses were done using the open-source statistical software package R (version 3.5¸https://www.r-project.org/).

We expected group differences between AP and RP regarding AQ scores, MAD (PAT), PIS (sum of correctly identified tones), and network parameters *γ* and *λ* (in beta, delta, and theta band). Additional unexpected results obtained in other frequency bands and network parameters are also reported. In order to correct for multiple comparisons across frequency bands, ten thresholds each, and various network parameters, only significant results within at least two successive thresholds were considered significant. We are aware that the common procedure to correct for multiple comparisons would be the application of false discovery rates or similar. However, we did not consider this reasonable for the following arguments: First, there is no clearly defined consensus on the number or level of thresholding. Second, it is not entirely clear how successive thresholds relate to each other and resulting graph theoretical measures [59] and if they can be assumed independent. The latter seems unlikely as the next higher density was always retrieved in taking the smaller network and adding connections. Third, small-worldness cannot be viewed independent from path length and clustering as its calculation depends on both of them (see the “Network analysis—graph theory” section). Thus, our approach mimicked a cluster-based approach whereby we only considered results significant if they replicated in at least two successive thresholds [101]. Results were obtained using *t* tests and non-parametric equivalents when applicable. Intercorrelations between the variables were investigated to further explore the interrelation of autistic traits, absolute pitch performance, and network structure using regression and bivariate correlations. Finally, the network parameters *λ* and *γ*, the AQ score, and the age of beginning to play a musical instrument (as a covariate) were used to predict PIS and PAT performance within the sample using multiple regressions and AQ and AP performance to predict network parameters.

## Results

### Behavioral performance and autism traits

The Welch two-sample *t* tests revealed significant lower absolute deviations from target tone (MAD; *t*(43.7) = 15.614; *p* < 2.2e−16) and lower deviations from individual mean deviation, i.e., interpreted as pitch template (SDfoM; *t*(40.9) = 12.145; *p* = 3.788e−15) for absolute pitch compared to relative pitch possessors (Table 2). Having AP was further associated with more autistic traits (AQ; *t*(60.3) = − 2.501; *p* < 0.015) and (marginally) an early start of musical training (starting age; *t*(55.4) = 1.751; *p* < 0.086). For AQ, only the subscale “imagination” reached significance (*t*(57.4) = − 4.287, *p* < 6.997e−05) with higher values for AP, while “communication” (*t*(55.3) = − 1.977, *p* = 0.053) and “attention to detail” (*t*(61.6) = − 1.776, *p* = 0.081) were marginal and “social skills” (*t*(60.9) = − 1.145, *p* = 0.257) and “attention switching” (*t*(62.0) = 1.012, *p* = 0.316) were not significant.

**Table 2:** Group differences

	AP (*n* = 31)			RP (*n* = 33)			*t* test*
	Mean	SD	Range	Mean	SD	Range	
AQ	20.48	6.05	10–36	16.88	5.44	6–27	*t(60.3) = − 2.501; p = 0.015*
MAD	41.37	36.49	9.8–200.57	296.84	86.12	91.04–467.52	*t(43.7) = 15.614; p < 2.2e−16*
SDfoM	52.31	44.96	7.41–235.69	329.77	122.77	134.37–811.73	*t(40.9) = 12.145; p = 3.788e−15*
Starting age	5.97	2.97	2–17	7.12	2.22	3–12	*t*(55.4) = 1.751; *p* = 0.086

Age, nonverbal IQ (SPM), information processing capacity (ZVT), musical training (total hours during life span on main instrument), musicality (AMMA; MSI), and online pitch identification screening (PIS) for each group.

*One RP has reported himself not having an absolute pitch but reached a screening score of 13. Because of this and the weak performance in the pitch adjustment test, the subject was assigned to the RP group. Significant group differences are indicated in italics. Welch two-sample *t* test

### Network analysis

**Fig. 1:**
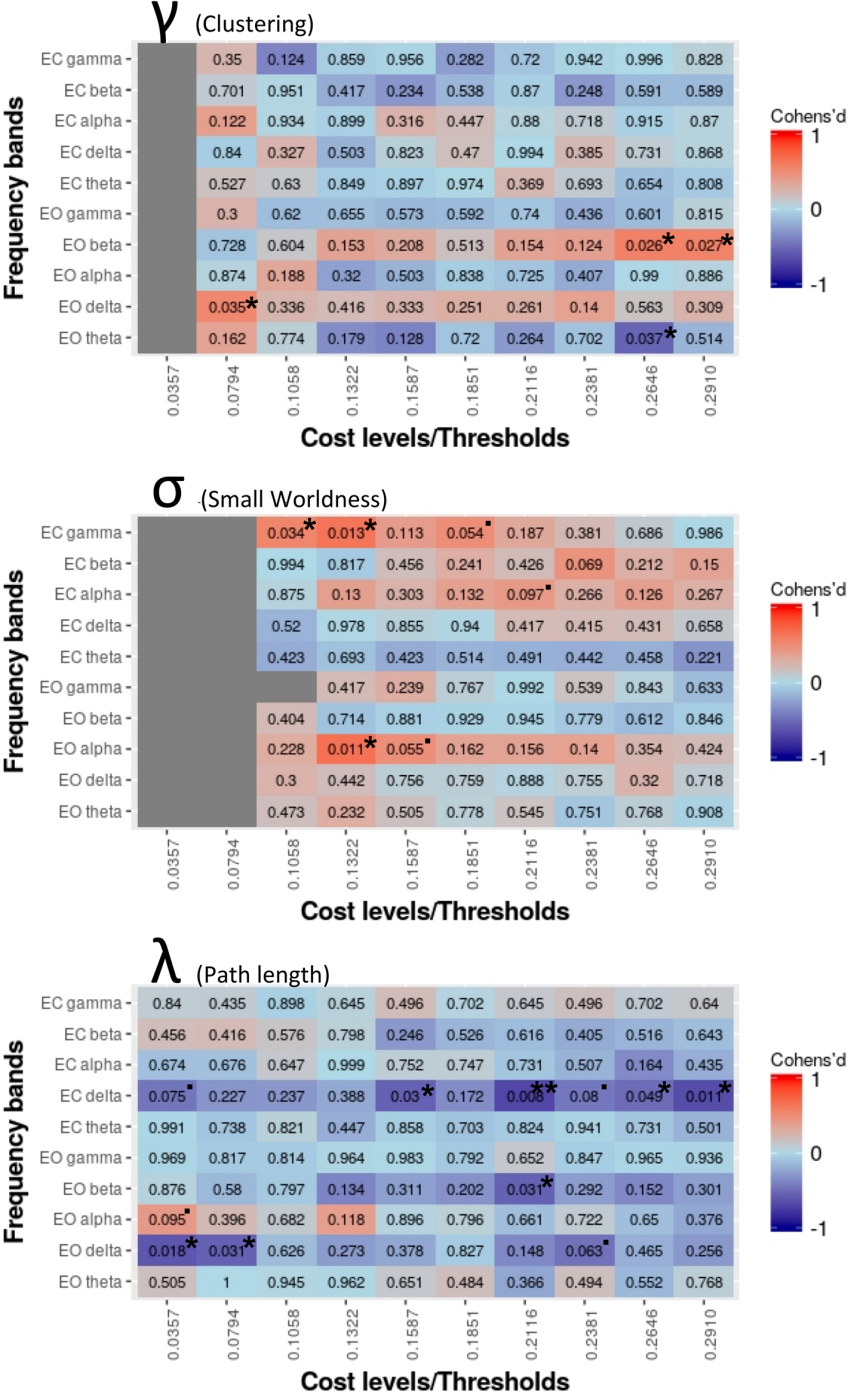
Multiple comparisons (Welch two-sample *t* tests) across frequency bands, thresholds, and eyes closed vs. eyes open RS between AP and RP. Matrix cells contain *p* values (uncorrected) and are colored according to Cohen’s *d* values. Blue cells indicate higher SW (small-world), Lrand (path length compared to random network) and Crand (clustering compared to random network) for AP compared to RP. Red cells show higher parameters for RP. Significant results (*p < 0.05; **p < 0.01; ***p < 0.001) and tendencies (“.”*p* < 0.10) are marked

The Welch two-sample *t* tests (*p* < 0.05, uncorrected) revealed higher average path length *λ* for AP compared to RP within the delta band (2–4 Hz) for both, eyes open (EO) and eyes closed (EC), resting-state conditions and at least two thresholds each. Lower path length values for AP were found in alpha (7–13 Hz) and beta (13–18 Hz) eyes open condition for one threshold each but did not reach significance (*p* < 0.10; see Fig. 1). Analysis of clustering coefficient *γ* yielded lower clustering for AP in EO delta (*p* < 0.05) for one threshold and EO beta (*p* < 0.10) for two neighboring thresholds. RP exhibited higher clustering for a single threshold in EO theta (*p* < 0.10). Small-worldness σ was widely reduced in AP within EC gamma, EC alpha, and EO alpha with significant (*p* < 0.05) or marginally significant (*p* < 0.10) group differences across one or two thresholds each (Fig. 1). No significant higher thresholds were found for AP.

In general, significant and marginally significant results were spread widely across different thresholds (see Fig. 1). Only significant results appearing on at least two thresholds in the same frequency band were included for further analyses (multiple regression). Of those, the threshold (*T*) with the highest effect size of neighboring significant results was taken: clustering *γ* EO beta (*T* = 0.2910), small-worldness σ EC gamma (*T* = 0.1322), and path length *λ* EC delta (*T* = 0.2116). Path length EO delta (*T* = 0.0357) was not taken into account because of correlation with path length EC delta (*T* = 0.2116).

### Prediction of absolute pitch performance

Multiple regression analysis was used to predict AP performance in pitch naming (PIS) and pitch adjustment (PAT). A multiple regression predicting PIS performance by autistic traits (AQ; beta = 0.892, *p* < 0.0001), clustering (C_EO_b10; beta = − 66.074, *p* < 0.0002), path length (L_ECd10; beta = 76.909, *p* < 0.008), and small-worldness (ECg4; beta = − 6.612, *p* < 0.0325) explained 44% of the variance (*R*^2^ = 0.44, *R*^2^_adjusted_ = 0.401; *F*(4,57) = 11.22, *p* < 9.92e−17). PAT performance was predicted by the same predictors plus the age of beginning of musical training (starting age) and explained 38% of the variance (*R*^2^ = 0.380, *R*^2^_adjusted_ = 0.326; *F*(5,57) = 6.991, *p* < 3.736e−05). Here, AQ (beta = − 0.089, *p* < 0.004), clustering (beta = 6.775, *p* < 0.004), and small-worldness (beta = 0.946, *p* < 0.023)) significantly contributed to the prediction, while the age of the beginning of musical training (beta = 0.130, *p* < 0.053) and path length (beta = − 7.006, *p* < 0.070) remained marginally significant. Bivariate pearson correlations among the variables are listed in Table 3. Post hoc mediation analysis revealed no significant mediation of the influence of network structure (graph theoretical parameters) on absolute pitch performance (MAD) by autistic traits (*p* > .05 for all comparisons).

**Table 3:** Bivariate correlations between variables of interest

Correlation coefficient (Pearson)								
*p* value	PIS	0.38**	− 0.91***	− 0.85***	−0.23	0.35**	−0.30*	− 0.28*
	0.002**	AQ	− 0.28*	− 0.25*	0.025	0.13	0.20	0.022
	< 0.001***	0.024*	MAD^a^	0.93***	0.30*	−0.27*	0.28*	0.31*
	< 0.001***	0.045*	< 0.001***	SDfoM^a^	0.25	−0.21	0.25	0.21
	0.074 .	0.844	0.017*	0.053	Start age	0.018	0.22	0.10
	0.005**	0.315	0.033*	0.094	0.887	*λ* EC delta	0.08	−0.23
	0.017*	0.109	0.026*	0.051	0.079	0.534	*γ* EO beta	−0.044
	0.028*	0.866	0.013*	0.100	0.431	0.075	0.731	*σ* EC gamma

Pearson correlations between variables of interest (network parameters: selected bands and thresholds)

*Significant correlation coefficients

^a^Variables were z-standardized to the mean and SD of the non-AP population

### Prediction of network parameters

To further investigate the interrelation between AP, autistic traits, and network connectivity, we calculated the general linear models to predict network connectivity (L, C, SW) differences obtained before by a combination of AP performance and AQ. Different models were compared using *R*^2^, *R*^2^_adjusted_, and information criteria (AIC). Separate models are shown for active (PAT) and passive (PIS) AP performance as for their high collinearity. Only clustering obtained a better prediction by a joint model of AQ and AP performance (active and passive on separate models because of intercorrelation) with AQ as a significant predictor. While the inclusion of AQ scores did not improve the prediction of path length and small-worldness (see Table 4), it was predictive for clustering coefficients in the beta range in each joint model with either MAD (*F*(2,60) = 6.011, *p* < 0.004; *R*^2^ = 0.167, *R*^2^_adjusted_ = 0.139; *β*_AQ_ = 4.06e−3, *p* < 0.014; *β*_MAD_ = 2.07e−4, *p* < 0.004) or PIS performance (*F*(2,59) = 6.889, *p* < 0.002; *R*^2^ = 0.189, *R*^2^_adjusted_ = 0.162; *β*_AQ_ = 4.44e−3, *p* < 0.009; *β*_MAD_ = − 2.62e−3, *p* < 0.0041). Both models were superior compared to a prediction of network connectivity by AP performance alone, even though the bivariate correlation between AQ and clustering did not reach significance (see the previous section). Post hoc mediation analysis revealed no significant mediation of the influence of absolute pitch ability (MAD) on network parameters by autistic traits (*p* > .05 for all comparisons).

**Table 4:** Comparison of models predicting network parameters by AP and AQ

		Predictors (*β*)					Comparison of models		
*γ* EO beta	Intercept	MAD	PIS	AQ	*F* (df)	*p* value	*R* ^2^	*R* ^2^_adjusted_	AIC
Model 1	3.54e−1***	2.07e−4**	–	4.06e−3*	6.011 (2,60)	< 0.004**	0.167	0.139	− 145.45
Model 2	4.383–1***	1.58e−4*	–	–	5.232 (1,60)	< 0.026*	0.078	0.064	− 141.13
Model 3	4.25e−1***	–	− 2.62e−3**	4.44e−3**	6.889 (2, 59)	< 0.002**	0.189	0.162	− 146.01
Model 4	4.96e−1***	–	− 1.83e−3*	–	6.009 (1,60)	< 0.017*	0.091	0.076	− 140.91
*σ* EC gamma	Intercept	MAD	PIS	AQ	*F* value	*p* value	*R* ^2^	*R* ^2^_adjusted_	AIC
Model 1	5.4e−1*	1.02e−3*	–	5.25e−3	3.378 (2,60)	< 0.041*	0.101	0.071	74.09
Model 2	6.49e−1***	9.57e−4*	–	–	6.504	< 0.013*	0.096	0.081	72.43
Model 3	8.08e−1***	–	− 1.11e−2*	9.30e−3	2.981 (2,59)	< 0.058	0.092	0.061	73.53
Model 4	9.56e−1***	–	− 9.41e−3*	–	5.06 (1,60)	< 0.028*	0.078	0.062	72.48
*λ* EC delta	Intercept	MAD	PIS	AQ	*F* value	*p* value	*R* ^2^	*R* ^2^_adjusted_	AIC
Model 1	1.82***	− 8.34e−5	–	4.40e−04	2.433 (2,60)	0.096	0.075	0.044	− 205.34
Model 2	1.83***	− 8.88e−5*	–	–	4.736 (1,61)	< 0.033*	0.072	0.057	− 207.14
Model 3	1.79***	–	1.30e−3**	− 1.29e−5	4.228 (2,59)	< 0.019*	0.125	0.096	− 204.45
Model 4	1.79***	–	1.29e−3**	–	8.6 (1,60)	< 0.005**	0.125	0.111	− 206.45

Parameters, significance (*F* statistics), and comparison of different models. Models are compared using *R*^2^, *R*^2^_adjusted_, and AIC (Akaike information criterion). Smaller AIC and higher *R*^2^ indicate superior models. *p < 0.05, **p < 0.01, ***p < 0.01 (uncorrected)

### Post hoc analysis: single connection statistics

**Fig. 2:**
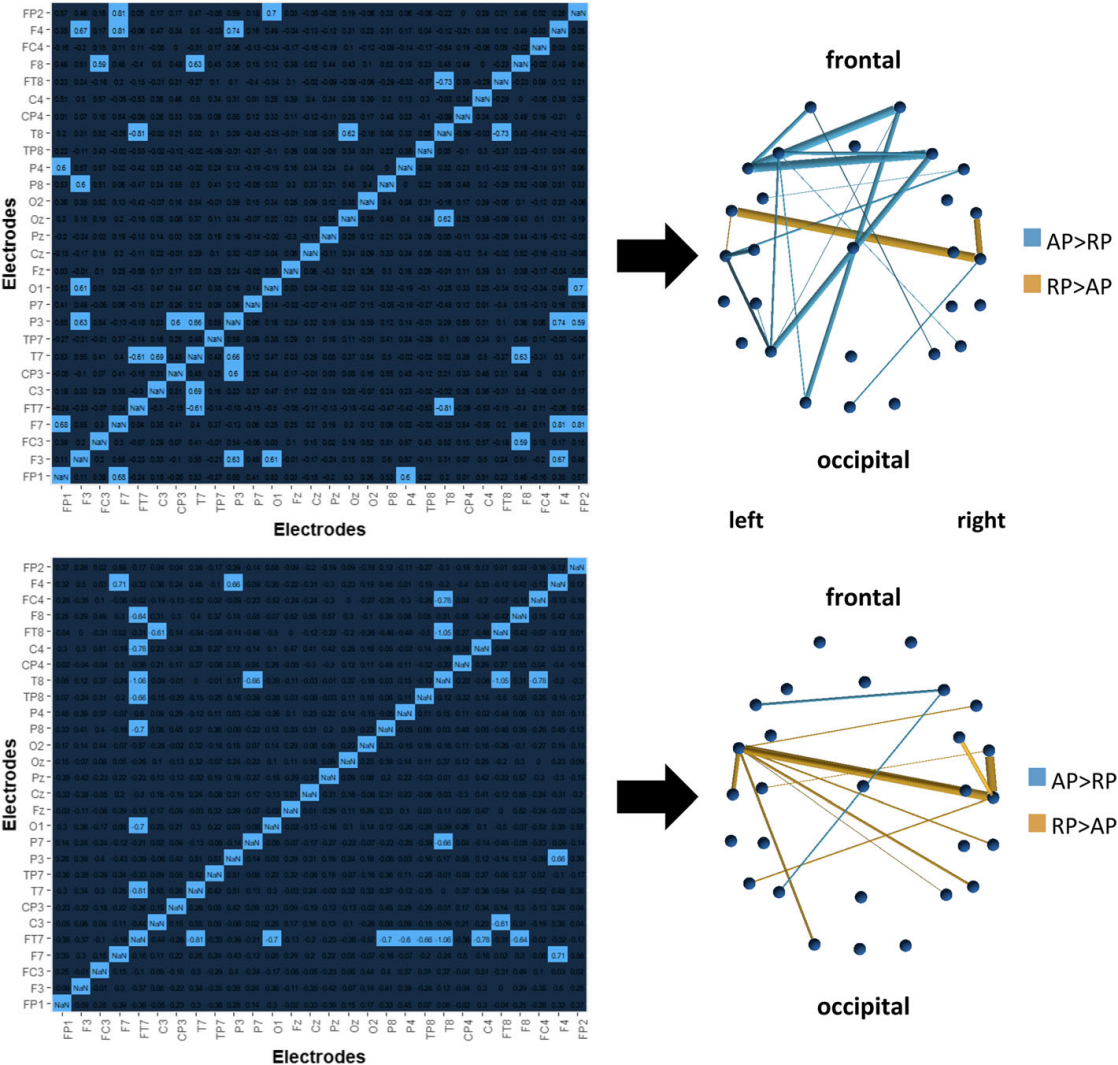
Visualization of single connection differences in the beta range. *Left:* Cohen’s *d* effect size values for all pairs of electrodes between groups in separate matrices for unstandardized (top) vs. z-standardized (bottom) raw connectivity matrices (permutation testing). Significant connections (FDR corrected) are highlighted in light blue. *Right:* Significant differences plotted in EEG-cap order (extended 10–20 system, view from above). Colors indicate the direction of effect (blue: AP > RP, yellow: RP < AP) and size of the line corresponding the effect size (Cohen’s *d*)

To assess single connection differences in the beta frequency band, permutation statistics (*n*_permutations_ = 10,000) across groups were evaluated post hoc. To obtain these, raw matrices in the relevant frequency bands (significant results) were z-standardized individually and permutation group statistics (FDR corrected) performed across groups using custom MATLAB scripts. An unstandardized comparison was provided as well. While the former reflects the relative importance of the connections within the participants’ networks between the groups, the latter shows group differences in the absolute wPLI. Results revealed overall increased wPLI values for AP in a network comprising mainly the left frontal and parietal regions (especially nodes F7, F3, F4, P3; see Table 5 for anatomical correlations) combined with lower connectivity within and between the bilateral temporal regions (FT7–T8, FT7–T7, FT8–T8; unstandardized results). Relative to their own networks (z-standardized participants matrices), APs exhibited a reduced connectivity compared to RP between the left FT7 and various sites along frontal-temporal-occipital electrodes (F8, T8, TP8, P8, P3) in the right hemisphere, especially again within and between the bilateral temporal regions (FT7–T8, FT7–T7, FT8–T8). The only significant higher connections relative to their own network for AP were found between F7, F8, and P7. Figure 2 (brain nets created using the MATLAB toolbox BrainNet Viewer [111]) shows Cohen’s *d* effect size values for all pairs of electrodes between groups in separate matrices for z-standardized vs. unstandardized raw connectivity matrices. The most pronounced differences that were found in both standardized and unstandardized (relative) comparisons comprise a reduced interconnection between bilateral auditory cortices (FT7–T8, FT7–T7, FT8–T8) as well as higher frontal-parietal connectivity (F7–F8, F8–P7) for AP. These connections therefore not only exhibit a group difference on absolute wPLI values, but also play a different role relative to the other connections in the participants’ networks.

**Table 5:** Cranio-cerebral correlations for electrode positions (10–10 system, modified after [110])

Electrode label	Talairach coordinates (mm)			Anatomical region		
	*x*	*y*	*z*	Lobe	Gyri	BA
FP1	− 21.2 ± 4.7	66.9 ± 3.8	12.1 ± 6.6	L FL	Superior frontal G	10 (100%)
FP2	24.3 ± 3.2	66.3 ± 3.5	12.5 ± 6.1	R FL	Superior frontal G	10 (100%)
F3	− 39.7 ± 5.0	25.3 ± 7.5	44.7 ± 7.9	L FL	Middle frontal G	(75%), 6 (19%), 46 (6%)
F4	41.9 ± 4.8	27.5 ± 7.3	43.9 ± 7.6	R FL	Middle frontal G	8 (69%), 6 (6%), 9 (25%)
FC3	− 45.5 ± 5.5	2.4 ± 8.3	51.3 ± 6.2	L FL	Middle frontal G	6 (75%), 4 (12.5%), 8 (12.5%)
FC4	47.5 ± 4.4	4.6 ± 7.6	49.7 ± 6.7	R FL	Middle frontal G	8 (69%), 6 (6%), 9 (25%)
C3	− 49.1 ± 5.5	− 20.7 ± 9.1	53.2 ± 6.1	L PL	Postcentral G	21 (62.5%), 22 (25%), 20 (6.5%), 42 (6%)
C4	50.3 ± 4.6	− 18.8 ± 8.3	53.0 ± 6.4	R PL	Postcentral G	123 (81.5%), 6 (12.5%), 40 (6%)
CP3	− 46.9 ± 5.8	− 47.7 ± 9.3	49.7 ± 7.7	L PL	Inferior parietal G	40 (82%), 123 (6%), 5 (6%), 39 (6%)
CP4	49.5 ± 5.9	− 45.5 ± 7.9	50.7 ± 7.1	R PL	Inferior parietal G	40 (77.5%), 123 (12.5%)
P3	− 41.4 ± 5.7	− 67.8 ± 8.4	42.4 ± 9.5	L PL	Precuneus	39 (37.5%), 7 (25%), 19 (25%), 40 (12.5%)
P4	44.2 ± 6.5	− 65.8 ± 8.1	42.7 ± 8.5	R PL	Inferior parietal L	39 (31%), 7 (25%), 40 (25%), 19 (19%)
F7	− 52.1 ± 3.0	28.6 ± 6.4	3.8 ± 5.6	L FL	Inferior frontal G	45 (56%), 47 (38%), 46 (6%)
F8	53.2 ± 2.8	28.4 ± 6.3	3.1 ± 6.9	R FL	Inferior frontal G	45 (37.5%), 47 (37.5%), 46 (25%)
FT7	− 59.2 ± 3.1	3.4 ± 5.6	− 2.1 ± 7.5	L TL	Superior temporal G	22 (75.5%), 21 (12.5%), 38 (6%), 44 (6%)
FT8	60.2 ± 2.5	4.7 ± 5.1	− 2.8 ± 6.3	R TL	Superior temporal G	22 (75%), 21 (13%), 38 (6%), 44 (6%)
T7	− 65.8 ± 3.3	− 17.8 ± 6.8	− 2.9 ± 6.1	L TL	Middle temporal G	21 (81.5%), 22 (12.5%), 43 (6%)
T8	67.4 ± 2.3	− 18.5 ± 6.9	− 3.4 ± 7.0	R TL	Middle temporal G	4 (50%), 123 (25%), 6 (25%)
TP7	− 63.6 ± 4.5	− 44.7 ± 7.2	− 4.0 ± 6.6	L TL	Middle temporal G	21 (50%), 37 (25%), 22 (19%), 20 (6%)
TP8	64.6 ± 3.3	− 45.4 ± 6.6	− 3.7 ± 7.3	R TL	Middle temporal G	21 (62.5%), 22 (12.5%), 20 (12.5%), 37 (12.5%)
P7	− 55.9 ± 4.5	− 64.8 ± 5.3	0.0 ± 9.3	L TL	Inferior temporal G	37 (44%), 19 (38%), 39 (18%)
P8	56.4 ± 3.7	− 64.4 ± 5.6	0.1 ± 8.5	R TL	Inferior temporal G	19 (56%), 37 (19%), 20 (12.5), 39 (12.5%)
O1	− 25.8 ± 6.3	− 93.3 ± 4.6	7.7 ± 12.3	L OL	Middle occipital G	18 (81%), 19 (19%)
O2	25.0 ± 5.7	− 95.2 ± 5.8	6.2 ± 11.4	R OL	Middle occipital G	18 18 (81%), 19 (19%)
Oz	0.3 ± 5.9	− 97.1 ± 5.2	8.7 ± 11.6	M OL	Cuneus	18 (62.5), 17 (31%), 19 (6.5%)
Fz	0.0 ± 6.4	26.8 ± 7.9	60.6 ± 6.5	M FL	Bilateral medial	6 (81.5%), 8 (12.5%), 9 (6%)
Cz	0.8 ± 4.9	−Ȁ921.9 ± 9.4	77.4 ± 6.7	M FL	Precentral G	4 (62.5%), 6 (37.5%)
Pz	0.7 ± 6.3	− 69.3 ± 8.4	56.9 ± 9.9	M PL	Superior parietal L	7 (88%), 5 (6%), 19 (6%)

Estimated projection of electrode positions to cortical areas (Talairach space) and variability of associated BA (Brodman areas), investigated by [110] using EEG-MRI sensors

*L* left, *R* right, *FL* frontal lobe, *PL* parietal lobe, *TL* temporal lobe, *OL* occipital lobe, *L* lobe, *G* Gyrus

Rough anatomical associations of electrode positions, taken from Koessler et al. [112], are summarized in Table 5. However, it must be clearly said that graph theoretical accounts and single connection permutation tests are completely different techniques and cannot be compared directly. This is because in the course of graph theoretical analysis, thresholds have to be applied on the participants’ raw matrices, leading to a reduced number of total connections. Thus, the connections fed into the graph analysis also highly depend on the participant-specific order of connection weights and can have a high regional variability despite producing similarly high or low network parameters.

## Discussion

The results of the present study underline a possible interrelation between autistic traits, brain connectivity, and absolute pitch ability. We investigated the EEG-resting state connectivity using a graph theory approach in professional musicians with and without absolute pitch, the Autism Spectrum Quotient [80], and a test of pitch naming and pitch adjustment ability. The analyses revealed higher autistic traits, higher average path length (delta 2–4 Hz), lower average clustering (beta 13–20 Hz), lower small-worldness (gamma 30–60 Hz), and a tendency for an earlier start of musical training in absolute pitch musicians. Furthermore, pitch naming was well predicted by autistic traits, path length, and clustering values, explaining a total of 44% of the variance. Pitch adjustment (i.e., active absolute pitch) was explained by the same predictors plus the age of the beginning of musical training summing up to an *R*^2^ = 0.38. However, in the latter case, the starting age of musical training and path length remained marginally significant.

It is noteworthy that the start of playing a musical instrument in our models did not significantly improve the prediction of AP performance but only in pitch adjustment. Furthermore, the total amount of musical training during life neither was predictive of any AP performance in the general linear model, nor did show a group difference. The typical human brain exhibits a small-world-like structure with a much higher clustering compared to a random network, while maintaining an efficient information transfer and low wiring cost through an equally low path length [70, 103, 107]. In this context, the results of the present study indicate a less efficient and less small-world structured functional network in AP compared to RP, in line with the structural results of Jäncke et al. [49] and the results from the autism research [52, 53, 56, 100, 113] but extends the results to EEG functional connectivity networks.

It is further interesting that both correlations and regressions between autistic traits and the two AP tests show higher correlations and better prediction of pitch naming than pitch adjustment by AQ. This can be explained by the aforementioned theory of veridical mapping [7, 69]. This framework explains savant abilities and other unusual abilities in autism by their common characteristic of one-to-one mappings between elements of two conceptual or perceptual structures (e.g., letters-musical tones, letters-colors). According to this theory, all of these abilities share further commonalities including hyper-systemizing [61]and enhanced perceptual functioning [59, 60], and depend on the exposure to material, and—if they occur as autistic savant ability—the related elements can also be recalled without a strategy [7, 69]). This explicit recall in the absolute pitch, i.e., the naming of the pitch, therefore might be a more savant-like ability, leading to a higher correlation with autistic traits.

Furthermore, we observed a reduced connectivity for AP compared to RP in interhemispheric connections when compared to the participants own distribution of connectivities (z-standardized calculation)—especially between the left auditory-located electrodes and various right temporal, parietal, and frontal electrodes.

While higher path length in low frequency bands (delta, therefore reduced integration) and lower clustering in higher frequencies (beta, reduced segregation on sensor level) are in line with our a priori hypotheses, we did not expect the reduced small-worldness within the gamma band for AP compared to RP (found during eyes closed). Nevertheless, this result can be explained by previous research findings: Cantero et al. [114] reported an increased gamma band measured by intracranial electrodes between hippocampal areas and neocortex in humans during wakefulness but not during sleep, pointing to a relation of gamma band couplings and awareness states in humans. This also suggests that gamma band activity, probably useful for the storage and retrieval of memory [115–117] and binding of perceptual features [116, 117], might even play a role during resting (awake more than asleep) states. AP ability, similarly, is often described as the ability to associate tones and verbal labels in a stable, hyper-memorized way, pointing to the importance of long-term memory processes [118–122]. Furthermore, Bhattacharya et al. [123, 124] found increased long-range gamma synchronization between distributed cortical areas during music listening in musicians compared to non-musicians, which might reflect musical memory and binding of musical features. In contrast, Sun et al. [125] found reductions in gamma-band phase locking and power in participants with autism associated with perceptual organization tasks (visual), while Brown et al. [126] found higher gamma peaks in response to illusory figures in autism. Generally, abnormal gamma activity is found in a range of neuropsychiatric disorders, with reduced gamma in negative schizophrenic symptoms, Alzheimer’s disease, and task-specific gamma decrease in autism, but an increase in gamma in ADHD, positive schizophrenic symptoms, and epilepsy (for a review see [127, 128]). Thus, the results of reduced small-worldness in AP are in line with an integration-deficit hypothesis of AP, both in perceptual organization and binding of musical stimuli and in brain connectivity, which is again similar to autism (see [50, 52, 129–132]). However, the findings in gamma band did not show correlations with autistic symptoms.

Our results replicate the results of Dohn et al. [47] showing higher autistic traits, which reached significance in the subscales “imagination” (similar to [47]), “attention to detail” (marginally), and “social skills” (marginally). Furthermore, autistic traits were also correlated not only to pitch naming as already shown by Dohn et al. [47], but also to pitch adjustment accuracy (MAD, mean absolute deviation to target tone in cent; 100 cent = 1 semitone) and adjustment consistency (SDfoM, pitch template tuning). However, similar to [47], the group mean autistic traits did not reach the cutoff for diagnostic relevance, indicating a high variability regarding autistic traits even in the AP group (with seven AP compared to one RP scoring above cutoff or borderline). This fits with the analyses of the broader autism phenotype [133] and might implicate joint as well as divergent phenotypic and endotypic characteristics of AP and autism.

In contrast to our study, various previous studies have shown an influence of the start of musical training in AP, making the onset of training before the age of 7 necessary, but not sufficient to acquire absolute pitch [12, 16, 20–22, 41]. For example, Loui et al. [41] recently found that early onset of musical training was associated with an enlarged tract between pSTG and pMTG in the left hemisphere, but the degree of AP proficiency still correlated with the size of the tract after partialling out the age of onset. Gregersen et al. [12] further analyzed the familiar aggregation of AP in different samples of musicians and non-musicians with early and late onset of musical training comparing different types of musical education and found no general differences of AP between early or late starting siblings of AP. Their results further indicated a higher influence of genetic disposition and the type of education used, which both had a more pronounced influence than the age of onset per se [12].

Higher average path length (delta 2–4 Hz)), lower average clustering (beta 13–20 Hz), and lower small-worldness (gamma 30–60 Hz) for AP compared to RP are also in line with previous studies showing structural local hyper- vs. global hypoconnectivity in AP [49] and reduced clustering and higher path length in participants with autism [113, 134]. In contrast, Loui et al. [51] reported overall increased degrees, clustering, and local efficiency coefficients of functional networks in AP using fMRI during music listening and rest. The authors further speculate that there might be a “dichotomy” between the structural and functional hyperconnectivity in AP, where the structure is locally hyperconnected but the function is globally hyperconnected [51]. The present study, however, provided more evidence for an also functionally underconnected brain in AP musicians compared to relative pitch musicians. Diverging results compared to Loui et al. [51] might be due to differences in methods (EEG vs. fMRI) or different definition of nodes (electrode positions vs. brain regions) and edges (wPLI vs. functional correlations).

Differences seen in single connection analysis might reflect the connections that lead to differences in clustering values described above. Similarly to the prediction of clustering by AP and autistic traits, single connection differences in the beta range are in line with the findings from the autism literature: First, various others have reported reduced interhemispheric connectivity in autism [56, 112, 113, 135, 136]. Second, hypoconnectivity between the left FT7 (BA:22) and right frontal-temporal-occipital electrodes (F8, T8, TP8, P8, P4; BA:45/47, 4, 21/22/20/37, 19/37, 39/7/40/19; see Table 4 for the anatomical interpretation of electrode positions) might reflect a specific underconnectivity between the left STG and right IFOF, of which alterations have already been described in both AP [137] and autism [138]. Especially reduced interhemispheric connectivity between the left auditory-related cortex and right IFOF might reflect autism-like personality traits and perception of (some) absolute pitch possessors. The IFOF, especially the right IFOF, has been shown to play an important role in music perception and the integration of musical features, as it connects various brain regions from the frontal over temporal to posterior parts of the brain [139]. A reduced white matter integrity of IFOF was found in amusics [139, 140], whereas people with synesthesia and absolute pitch were shown to have a higher IFOF integrity [67, 137]. More importantly, however, increased interhemispheric connectivity in musicians was found by several studies [141–145] showing the importance of interhemispheric integration in music perception. A reduced interhemispheric functional connectivity, especially between bilateral auditory regions as found in the present study, perhaps might result in less perceptual integration of musical features (i.e., auditory weak central coherence) and hence a more detail-oriented processing of music and musical pitches (i.e., absolute vs. relative) in those participants. An exaggeration of those features might also lead to symptoms of amusia, which has also been associated with alterations in the left and right STG and right IFOF [139, 140, 146] and with autism [147]. However, it must be clearly said that we cannot explicitly conclude the anatomical differences from connectivity differences on the sensor levels. Further structural or functional studies using methods with high anatomical precision have to be conducted to evaluate this hypothesis.

## Limitations

Some caveats of the present approach are warranted. First, we did not use a source-based approach of functional connectivity, making conclusions with respect to anatomical associations of the obtained differences very speculative. Second, various different configurations of local and global hyper- vs. hypoconnectivity can be assumed to result into the same averaged network measures; therefore, no conclusions can be made about the exact relative structure within the brain and among different regions. Nevertheless, higher path length (EC, delta 2–4 Hz) can be interpreted as weaker integration in the network and higher clustering (EO,13–20 Hz) as higher local segregation of functions [95] and therefore might again reflect a local hyper- over global (integrative) hypoconnectivity in the brain of AP musicians. This interpretation is further encouraged by studies showing that long-range connectivity (integration) is more reflected in low frequency bands, whereas short-range connectivity is more in high frequency bands [110, 148]. This again fits to the results of our study, as higher clustering, indicative for local segregation, was found in the beta range and path length—indicative for global integration in the network and therefore long-range associations—in the delta range.

Furthermore, evidence for a higher proportion of AP among people with autism or Williams syndrome, as mentioned in the introduction, is currently mainly based on case studies and case reports, as systematic epidemiologic studies have not been conducted yet and studies with respect to Williams syndrome are rare. Therefore, the actual co-occurrence between the phenomena remains to be evaluated. On top of that, the *R*^2^ values for predicting brain connectivity by AP and autism seem comparably weak. Mediation analysis did not reveal a mediating influence of autistic traits on the relation between absolute pitch and brain connectivity (both directions). However, the nature of altered brain connectivity is a common phenotype for numerous neuropsychiatric disorders and phenomena, not just autism and absolute pitch. Therefore, we have to admit that a range of other factors influencing brain connectivity would have been necessary to get a more detailed insight into what influences brain connectivity and vice versa. Most likely a relation between autistic traits and absolute pitch is only true for a subgroup of absolute pitch possessors. Bigger sample sizes are necessary to investigate this hypothesis.

In addition, significant group differences were highly selective for certain frequency bands, states (EO vs. EC), and thresholds. Nevertheless, we can rule out the possibility that we obtained those differences by chance. First, there were significant differences for at least one threshold in a frequency band, and effect sizes of the other thresholds in the same frequency band never (exclusive: Crand EO alpha) indicated reverse effects (see color code in Fig. 1). Second, we did only consider differences relevant if at least two neighboring thresholds exhibited a significant group difference. Third, the three network parameters selected via group differences always could also predict AP performance with a reasonable high *R*^2^ and/or showed bivariate correlations with AP performance in both tests of AP.

## Conclusion

For the first time, we included a pitch adjustment test of active absolute pitch [79] into a study on brain connectivity in AP, so we are not only referring to pitch naming as were previous studies [41, 47, 49, 51]. Also, whereas Jäncke et al. [49] were using structural cortical thickness covariations and Loui et al. [51] functional correlations of fMRI activity (during rest and music listening) as weights for connections in graph analysis, we for the first time applied graph theory on the resting-state EEG connectivity of AP musicians, both in eyes closed and eyes open conditions. This is similar to methods used in analyzing brain connectivity in autism [57, 113]. Finally, while Elmer et al. [118] used phase synchronization as an estimate for functional EEG connectivity, we used wPLI (weighted phase lag index, [85]), which is less contaminated by volume conduction [85–88, 91], thus contributing to a higher validity and reliability with respect to true brain connectivity and graph theoretical parameters [89, 93, 94].

In summary, differences in network and connectivity analysis in the beta band seem to be specifically associated with the relation of autistic traits and absolute pitch, whereas path length in delta range and small-worldness in gamma range might reflect other influences on the acquisition of the ability (e.g., environmental factors, genetic factors not attributable to autistic traits, musical education method, instrument, learning, sensitive periods). To our knowledge, this is the first study to combine measures on autistic traits and brain networks on musicians with and without absolute pitch. We conclude that this is further evidence showing that both AP and autism have shared and distinct neuronal and phenotypic characteristics. This might also be reflected in subgroups of AP with different genesis, providing new arguments for the discussion about a dichotomous or continuous view on AP. However, the causal relationship between AP, autistic traits, and brain connectivity remains to be evaluated.

## Abbreviations

σ: Small-worldness
AMMA: Advanced Measures of Music Audiation
AP: Absolute pitch
AQ: Autism quotient
ASD: Autism spectrum disorder or condition
C: Clustering coefficient
EC: Eyes closed resting state
EEG: Electroencephalography
EO: Eyes open resting state
GOLD-MSI: Musical Sophistication Index
ICA: Independent component analysis
imag: Imaginary component
L: Path length
MAD: Mean absolute derivation from target tone
MST: Minimum spanning tree
PAT: Pitch adjustment test
PC: Phase coherence
PIS: Pitch identification screening
RP: Relative pitch
SDfoM: Standard deviation from own mean
sgn: Sign
SPM: Standard Progressive Matrices
*S*_xyt_: Cross-spectrum
wPLI: Weighted phase lag index
ZVT: “Zahlenverbindungstest” (Trail Making Test)
*γ*: Clustering relative to random network of same cost and density distribution
*λ*: Path length relative to random network of same cost and density distribution
